# The Effect of Breastfeeding on Food Allergies in Newborns and Infants

**DOI:** 10.3390/children10061046

**Published:** 2023-06-12

**Authors:** Zoi Koukou, Eleftheria Papadopoulou, Eleftherios Panteris, Styliani Papadopoulou, Anna Skordou, Maria Karamaliki, Elisavet Diamanti

**Affiliations:** 1 School of Health Sciences, International Hellenic University (IHU), Sindos, 57400 Thessaloniki, Greece; stellao@hotmail.gr (S.P.); anna.lerks@gmail.com (A.S.); mariakaramaliki@gmail.com (M.K.); 2 Department of Pediatrics, University Hospital of Heraklion, 71500 Heraklion, Greece; 3 Laboratory of Forensic Medicine and Toxicology, School of Medicine, Aristotle University of Thessaloniki, 54124 Thessaloniki, Greece; panteris@chem.auth.gr; 4 School of Medicine, Faculty of Health Sciences, Aristotle University of Thessaloniki, 54124 Thessaloniki, Greece; diamantie@auth.gr

**Keywords:** breastmilk, colostrum, breast feeding duration, allergies

## Abstract

Breastfeeding is the preferred method of infant feeding and its establishment is one of the primary goals for the infant. Allergic diseases are common in childhood, with increased morbidity. Food allergies are also associated with a strong negative impact on health-related quality of life and is a major public health problem. In addition, maternal exclusion of common allergens during pregnancy and/or lactation suggests that supplementation with regular cow’s milk formula during the first week of life should be avoided. Breast milk contains many active immune factors, such as cytokines, inflammatory mediators, signaling molecules and soluble receptors, which may also reduce the risk of allergic disease. The prophylactic effects of breastfeeding have been the subject of many studies, some with weak evidence. In this narrative review, we aim to provide an up-to-date account of the effects of prophylactic breastfeeding on food allergy and other common allergies in infants and children up to 5 years of age. Colostrum in particular has been shown to be prophylactic against food allergy. The American Academy of Pediatrics cautions that the relationship between duration of breastfeeding and incidence of food allergy in early childhood is unclear. The protective role of breastfeeding has a positive effect on allergy prevention, which is opposed by the early introduction of solid foods, but larger studies are needed to confirm the evidence. There is evidence that breastfeeding is effective in providing partial protection to infants.

## 1. Introduction

Food allergies are caused by repeated exposure to a food substance that triggers a specific immune response that can lead to severe health effects such anaphylactic shock. During fetal life, a large array of food antigens cross the transplacental barrier and enter the body of the fetus, with the possibility of intrauterine sensitization [1,2]. Later, after birth, food allergens are differentiated according to the immunoglobulin response against target cells, with a high degree of sensitivity up to the first 24 months of life [1,3]. In addition, due to the unknown intrauterine or extrauterine sensitization of the fetus and later the infant, mothers continue their diet without specific hypoallergenic dietary instructions until symptoms appear [2,4]. Demographic data from the National Institute of Allergy and Infectious Diseases (NIAID) indicate that food allergies occur in 5% of children presenting with eczema, rhinitis and gastrointestinal problems [3], with increasing morbidity over the last 10–15 years [5].

Breastfeeding has been advocated as an solution to many a problem, from pain management in NICU infants [6], to negating some of the negative effects of the congenital Zika virus syndrome, that cause microcephaly [7].

Exclusive breastfeeding is essential for at least the first 6 months of life, with continued complementary breastfeeding for the first two years of the child’s life as per WHO guidelines [2,8].

## 2. Breastfeeding and Allergies

Breastmilk is immunologically the most relevant and essential food for the development of the newborn and the consequent maturation of immuno-defensive mechanisms [4,5]. It confers active and passive immunity, through maturation of the system and also provides bioactive components, like secretory IgA and IgG [5], with a proven prophylactic effect against food allergies [4]. In particular, colostrum contains the highest concentrations of bioactive components with immunological and anti-inflammatory activity. These are influenced by maternal and obstetric factors such as age, maternal diet and age of first pregnancy for the mother, while growth factor β (TGF-β) limits the development of allergies through the intestinal immune system [9]. It seems that breastmilk quality can be affected by the number of previous children [9]. In a very recent study, breastmilk samples were taken from 42 mothers to assess the levels and influence of TGF-1, TGF-2 (dominant type), and IgA in colostrum and, later, mature milk in primiparous and multiparous women [9]. TGF-1 and TGF-2 concentrations were greater in primiparous women, despite decreased colostrum production in the first hours. Furthermore, caesarean section seems to have a role in boosting TGF- factors secreted during breastfeeding due to the healing effects of the incision site, while postponing the start of lactation due to stress on the body [9]. Thus, it could be inferred that the next children of a primiparous mother are more likely to develop allergies in the future [9]. A contentious conclusion, given that a mother’s medical history, nutrition, stress, and stage of lactation all have an impact on the composition of milk at all phases [10].

The intestinal microbiota system is unique, as it strengthens the intestinal barrier through bacteria colonization, protecting the neonate immune system [4]. Due to immunological specialization, the manner and type of nutrition of the neonate have a significant impact on the gut-intestinal flora system [4]. Breastmilk is also known to provide the necessary foundation for safe colonization, so the relationship between the infant’s gut microbiota and the development of allergies may be affected [4].

Additionally, genetic factors control the release of anti-inflammatory and anti-allergic substances (immunoglobulins, oligosaccharides, growth, lysozyme, and lactoferrin) expressed by the mammary gland [4]. Of course, the main antigen transporter is not the permeability of the mammary gland, but most likely the intestinal transport of parental antigens, during milk production at the gland [4]. Due to its complexity and diversity, the anti-allergic effect of breastmilk is stronger compared to formula milk products [2], but is still at moderate level of evidence when compared within a meta-analysis of 17 studies. In infants, breastfeeding is associated with a reduction in asthma in later adulthood [3].

The most common form of food allergy in infants and young children under 3 years of age is sensitization to cow’s milk protein (casein or serum beta-lactoglobulin) [11]. The most common allergens are casein, alpha-lactalbumin, and beta-lactalbumin, the latter of which is extracted from breastmilk and is the trigger [12]. It has a 50–65% chance of being converted into an offending antigen, altering the intestinal flora and the concentration of bioactive compounds in the body [13]. The natural development of an infant’s gastrointestinal tract is essential for the metabolism of non-dairy foods, and its disruption can lead to future allergic manifestations [14]. It should be noted that only 0.5–1% of exclusively breastfed infants may later develop an allergy to cow’s milk protein [13] although there is a significant tendency to over diagnosis [15]. At the same time, it is difficult to diagnose because of its generic symptoms and is often differentially diagnosed as gastroesophageal reflux disease (GERD), abdominal pain, and gastrointestinal disorders [11]. The presence of blood in the stools (allergic proctocolitis [13]) immediately raises the suspicion of a food allergy, but in 65% of cases it is usually due to sensitivity to cow milk proteins [13,15].

The time of onset for allergic symptoms varies from within the first 2 min after ingestion to the first 48 h, with expression in the gastrointestinal and/or respiratory system and/or skin; the combination of systems implies IgE sensitization [11], whereas the later onset of symptoms up to 2 weeks after contact corresponds to eosinophil or T-cell sensitization [12]. The European Society for Paediatric Gastroenterology Hepatology and Nutrition (ESPGHAN) guidelines differentiate according to the severity of symptoms and the presence of breastfeeding. In particular, in breastfed infants with mild or moderate symptoms, overt and covert products containing cow’s milk protein are removed from the mother’s diet for 3 to 6 days; if delayed onset of symptoms is suspected, calcium supplementation is recommended for 2 weeks; while in severe symptoms (severe anemia), the mother’s milk is expressed and the newborn is given an improved anti-allergy formula [14]. In non-breastfed infants, animal protein sources and cow’s-milk-based formula should be removed. A hydrolyzed protein formula with proven results in this type of allergy is recommended. If this fails or is refused, either a soya formula (with proven tolerance) or an amino-acid-free formula is given to improve nitrogen supply [11,15].

The American Academy of Breastfeeding Medicine (ABM) protocol provides a universal protocol in case of allergic symptoms [13]. More specifically, it emphasizes the complete and correct recording of the family allergic medical history (for a parent or sibling) to determine the possibility of hereditary allergy by a qualified allergologist. The symptoms and their severity also create the need to assess the patient’s body weight, cardiac and respiratory status in order to choose a rapid or slow treatment modality. The ABM recommends the continuation of breastfeeding with the first step being the removal of cow’s milk derivatives (cheese, milk, yoghurt) from the mother’s diet [13]. In addition, foods with high allergenicity such as soy, chocolate, strawberries, nuts, and eggs are removed and attention should be paid to packaged products containing allergens for at least 2 to 4 weeks, with improvement within 72–96 h [13]. Otherwise, with severe symptoms, the withdrawn foods should be added back in weekly intervals and fish and gluten foods should be withdrawn as directed by a maternal dietitian with low-allergenic substitutes [13].

## 3. Breastfeeding and Its Effects on Asthma and Atopic Dermatitis

Breastfeeding and its beneficial effects have been of interest to the scientific community for years. The breastfeeding period is becoming one of the most critical and vulnerable periods in the life of newborns in terms of better development of the respiratory as well as other systems. Maternal nutrition plays an integral role in this phase [16].

A literature review study investigating asthma incidence and maternal breastfeeding as a pillar of antibody transfer has concluded that milk-transmitted allergens, IC and TGF-β, may induce tolerance to asthma in offspring, while inadequate exposure to milk-transmitted allergens or offspring exposure to a specific environmental allergen may promote asthma [4].

Breastmilk contains interleukin-7 (IL-7), which is known to be important for lymphocyte development. New studies have brought to light the fact that there is a positive correlation between the concentration of IL-7 in breastmilk and thymus development and lymphocyte production [17]. Cohort studies, including the WHEALS study, have concluded that breastfeeding is one of the most important factors in the development of the infant’s microbiome [4]. With regard to the above study, some of the models tested in mice did not seem to be applicable to humans, so the statistical results are poor. In particular, in mice, neonatal Fc receptors (FcRNs) function in the neonatal stage and transfer maternal IgG to milk through the gut epithelial barrier; however, in humans, FcRNs are highly active in transferring maternal IgG to the fetus across the placental barrier. Furthermore, human milk oligosaccharides have a more complex composition than mouse oligosaccharides. The IgA/IgG ratio is also different in human milk compared to mouse milk. These and other differences may have an impact on the ability to extrapolate findings from animal studies to mechanisms that function in a similar biological way [4]. The adoption of the Mediterranean diet has been proposed as an effective allergy-controlling treatment, especially in allergic proctocolitis [18] and in asthma [19].

In addition, some studies, such as the CHILD study, showed that breastfeeding is protective against asthma in the first 3 years of life [20]. Furthermore, a study was conducted in Qatar from 2006 to 2007 to explore the relationship between breastfeeding and newborn and later childhood allergic reactions. There were 1278 women who took part, along with their babies. They were divided into groups of breastfeeding and non-breastfeeding women. The study showed that breastfeeding was protective against respiratory diseases, unlike formula feeding [21]. Research has also shown that any form of breastfeeding, either shorter or longer duration, can and does work effectively in reducing eczema up to the age of 2 years [22].

In view of all the beneficial effects of breastfeeding, it is worthwhile to be aware of the potential effects that it may have. In particular, a number of allergic reactions can occur in infants as a result of maternal exposure to known allergens such as cow’s milk, egg, fish, gluten, peanuts and seeds [23]. Several studies have shown that, despite advice to limit and delay exposure to potentially allergenic foods, the incidence of food allergy is surprisingly increasing in high-income countries. While there is evidence of increased allergy risk when solids are introduced earlier than 3 or 4 months of age, there is no evidence that delaying the introduction of allergenic foods beyond 4 months of age, decreases the risk of allergies in infants in the general population, or, in those with a family history of atopic dermatitis [14].

A recent systematic review found that the early introduction of eggs at 4 to 6 months of age was associated with a reduced risk of egg allergy [24]. Two studies reported that infants first exposed to raw pasteurized egg may develop severe allergy due to prior sensitization. In addition, early introduction of peanuts at between 4 and 11 months of age was associated with a lower risk of developing peanut allergy [25]. The Paediatric Allergy Association has recommended that infants who are at high risk of developing a peanut allergy should be exposed to peanuts at an early age, following an appropriate expert assessment [14,23].

Additionally, a 2019 systematic review aimed to examine the association of formula and human breastmilk with food allergy, hay fever, allergic rhinitis, atopic dermatitis, and asthma. The review found that receiving little or no breastmilk was associated with an increased risk of childhood asthma. There was a limited amount of data on food allergy, allergic rhinitis and atopic dermatitis [3]. A similar study in 2019 attempted to correlate infant feeding with the development of allergies in early childhood [26]. The feeding regimens were divided into six groups: breastfeeding for 3 months then mixed feeding; breastfeeding for 3 months and formula followed by mixed feeding; breastfeeding for 1 month followed by mixed feeding; mixed feeding for 6 months, meaning simultaneous breastfeeding, formula and formula; immediate formula feeding for 2 to 3 months followed by formula and solids; and formula and solids from the first month.

Mixed-feeding infants had a higher risk of food allergy symptoms (risk ratio (RR) 1.54, 95% (CI) 1.04, 2.29) compared with 3 months breast-feeding. There was no statistically significant risk of the type of infant feeding for the diagnosis of food allergy by a physician. Prenatal maternal smoking was associated with an increase in the risk of food allergy diagnosis [26]. In conclusion, this study suggests that introducing multiple feeding sources may lead to food allergy symptoms; however, further research is needed to determine acceptable approaches to ameliorate childhood food allergy and the role of infant feeding [26].

Following the above studies, a new detailed study shows that late introduction of allergenic foods increases a child’s risk of developing allergies. However, there is no evidence to recommend introducing potentially allergenic foods prior to reaching six months of age [27]. Thus, while exposure to multiple food sources can cause allergic symptoms there is no conclusive evidence as to the appropriate treatment yet. Future efforts are needed to determine methods that can be used to identify food allergies in children and the role of infant feeding [26].

## 4. The Effects of Breastfeeding on the Development of the Gut Microbiome

The gut microbiome is an established homeostasis regulator, promoting baseline immune functionality and nutrient absorption for the whole body [28] and an active medicinal target [29,30]. In addition to affecting the well-being of the host, the gut microbiome plays an important role in several health-related aspects such as glucose tolerance, and diseases such as obesity, hyperinsulinemia and cardiovascular disease [31,32]

Diets high in fat induce intestinal microbiota disorders and are a cause of intestinal inflammation [30].

Breastfeeding and vaginal birth favourably influence the development of the infant’s gut microflora and protect against the development of allergies, as a recent study has confirmed. The intestinal microflora of breastfed infants is characterised by an early dominance of Bifidobacterium, which may have a significant impact on developing immune tolerance [33]. In addition to key macronutrients, breastmilk contains oligosaccharides, cytokines, chemokines and hormones, immunoglobulins, immune cells, active enzymes including peroxidases and lysozymes, lactoferrin and additional secretory components, TLR24 and soluble tumour necrosis factor (TNF) receptor, as well as food antigens from the maternal diet and the infant’s own microbiome, consisting of bacteria and viruses.

From the above data, it is clear that the increased risk of allergy in early childhood is indicative of an inadequate regulation of the immune system during this period of life [4]. Furthermore, the same study reported that breastfeeding by allergen-sensitised mothers may help prevent allergic diarrhoea and intestinal mast cell proliferation in the long term [4].

## 5. Colostrum and Allergies

Colostrum is the first food for newborns and has many important properties in the immune system. The main components of milk can be divided into three groups: bioactive factors, proteins and fats. The breastmilk of mothers of premature babies contains more protein than that of infant mothers [34]. From early lactation, breastmilk provides the newborn with about 108 maternal white blood cells per day. Most of the milk is made from milk oligosaccharides, which are not particularly beneficial but act as prebiotics to support the growth of prebiotic bacteria in the baby’s stomach.

Infants can develop allergies during the neonatal period as their immune system is regulated by T helper 2 cell (TH2) responses. As a result, the content of breastmilk is important and diverse, as it supports the child’s immune system and has factors that reduce allergies [35].

An in-depth study conducted in 2022 investigated colostrum and breastmilk and determined the percentage of different cell types by cytometry. The results showed a difference between the total number of cells in colostrum and breastmilk and the percentage of specific stem cells. At the same time, studies have shown that CD34+ cells in colostrum and high CD133+ cells and low CD105+ cells in colostrum are associated with an increased risk of developing eczema in the first 3 months of life. To our knowledge, this is the first study of the effect of stem cells on infant diseases and leads to a better understanding of human milk [35]. Stem cells are found in human milk and are more concentrated in human colostrum than in mature milk.

Interestingly, these cells are multipotent, and studies in mouse models have shown that human mammary stem cells can differentiate into many tissues, including the brain, thymus, pancreas, liver, spleen, and kidney [36].

A study on the immune response from colostrum and human milk have shown that the composition of antibodies such as Igs, LF, differ in human colostrum compared to cow’s colostrum [36]. Human colostrum is predominantly IgA, while bovine and porcine colostrum is predominantly IgG. Additionally, studies in premature infants have shown increased levels of proteins such as sIgA, LF, and α-lactalbumin in the blood and urine compared to breastfed and formula-fed infants. This suggests that the immature stomachs of premature infants can absorb large molecules such as Igs in colostrum and breastmilk [36].

## 6. Breastfeeding Duration and Effectiveness in Allergic Diseases

To reduce the incidence of allergy-related diseases in newborns in the short and long term, a controlled, prospective cohort study was conducted in Trondheim, Norway, in 2000 among pregnant women and their children [37]. The Norwegian recommendations were to breastfeed exclusively for the first six months of life and to continue breastfeeding until one year. Wheezing, asthma and eczema at 2 and 6 years of age were examined. The participants were women and children aged 6 weeks, 12 months, 1 year, and 2 years, divided into groups based on duration of breastfeeding. The findings showed that a longer duration of breastfeeding for at least 6 months was effective in reducing the incidence of eczema, asthma, and wheezing at the ages of 1, 2, and 6 years [37]. In a 2019 cohort study from New York State, the association of mode of delivery with the occurrence of respiratory wheeze and infant allergies to breastmilk was studied [38]. Infant wheezing was reported every 4 to 6 months until 3 years of age by questionnaire, as were food allergies beginning at 8 months. Breastfeeding conferred an association between mode of delivery and wheezing, but not food allergy [38].

In addition, the ARCO-kids study in Korea studied the effect of breastfeeding duration in a large cohort, involving 1374 children with rhinitis aged 4–12 years, showed that long-term breast feeding (≥12 months) was associated with a reduced risk of allergic rhinitis in Korean children [39].

Similar results were found in a Swedish study. Newborns who were breastfed for more than 4 months had a lower risk of eczema and allergic rhinitis at 4 years of age [40].

In another study from the United States, it was shown that prolonged breastfeeding in African American patients reduced the risk of allergic rhinitis at the age of 3 years [41]. Similarly a Spanish cohort study of 580 children followed from birth to 1 year and 2 months confirmed that exclusive breastfeeding for 4–6 months reduced wheezing, lower respiratory infections and eczema at 7–14 months [42]. Table 1 has the most important effects gathered together.

## 7. Introduction of Solid Foods and Food Allergies

The beginning of the introduction of foods other than breastmilk is a very important period for the infant, in particular it is a regulator of future eating behavior and is responsible for the future development of obesity, allergies, type 2 diabetes, cardiovascular disease, and metabolic activation [43]. The transitional period of food addition is called complementary feeding. It begins with a gradual reduction in breastfeeding and the simultaneous addition of solid foods to the infant’s meals [44]. After 6 months of exclusive breastfeeding until the first 2 years of life, breastfeeding is considered by the WHO to be complementary and adjunctive [45].

According to the European Society for Paediatric Gastroenterology, Hepatology and Nutrition (ESPGHAN), solid foods should be introduced at 4–6 months concurrently with breastfeeding, with a strong recommendation to start cow’s milk after the first year [14]. The recommended guideline for complementary feeding mentions the addition of small amounts, without replacing breastfeeding, for a smoother transition later on. Regarding the occurrence of food allergies in breastfed infants with complementary oral feeding, ESPGHAN guidelines insist on the sequence of removing allergenic foods (egg, fish, gluten, peanuts, and seeds) in the mother’s diet. In general, the cause-and-effect relationship between the onset of allergy and the introduction of solid foods is attributed to their early introduction, i.e., before the first 4 months or before 19 weeks [14,43].

Pre-existing sensitization during breastfeeding is also thought to cause an allergic reaction to solid foods. For example, newborns who developed symptoms during breastfeeding due to maternal egg consumption were likely to relapse when introduced to direct feeding. Therefore, the possibility of re-sensitization (mainly to the raw and/or unbleached product) is imminent when foods that previously caused problems are gradually introduced between 4 and 11 months [14,43].

The European Academy of Allergy and Clinical Immunology (EAACI) guidelines mention the prevention of the production of immunoglobulin E (IgE), which is responsible for the appearance of symptoms from a possible food allergy [2]. The most well-known allergens that affect a large number of the population include hen’s eggs, cow’s milk, and peanuts. For example, in the case of eggs, the recommendation is the addition in small quantities (½ egg) twice a week from the 4th month onwards, provided that it is not raw, since unpasteurized or raw eggs are dangerous, increasing the likelihood of an anaphylactic reaction. Regarding the introduction of peanuts, they should initially be administered in a safe form (e.g., melted peanut as peanut butter) without pressure, with slow administration so as not to block the airways during the test [2]. However, by 9 months of age most infants can use their hands to feed themselves small pieces or a cup for thicker meals in time [14]. In countries with a high prevalence of such allergies due to national cuisine, the health professional may recommend its introduction from 4 to 11 months while following local guidelines [2].

The evidence is clear that mothers should be encouraged to avoid certain foods during breast-feeding which may potentially be allergens in infants. Breastfeeding can be effective in the partial immunity of the offspring [2,11,14]. A correct and balanced diet contributes to normalizing and facilitating both mothers’ and infants’ quality of life. In particular, it is necessary for breastfeeding mothers to avoid all dairy products in their diet, while at the same time, if the infant receives any complementary foods or medicines, these need to be free of dairy products [2,11,14].

Breastfed infants with severe symptoms (e.g., severe atopic dermatitis or eczema or allergic (enteric) colitis complicated by stunting and/or hypoproteinemia and/or severe anemia) may be given therapeutic formula for several days to 2 weeks. Breastfeeding can be continued as normal after a follow-up visit to a specialist physician, so that appropriate counselling can be given to the mother [2,11,14].

Table 2 has the guidelines and recommendations from all the relevant societies on solid food introduction while breastfeeding.

Introducing gluten to the mother’s diet during breastfeeding may reduce the risk of not only celiac disease, but also type 1 diabetes and wheat allergy, according to a 2017 systematic review [14]. Next, it must be emphasized that in the first weeks after gluten introduction and during infancy, the consumption of large amounts of gluten should be avoided [46]. The same study showed that in children at high risk of type 1 diabetes, gluten introduction at <3 months of age was associated with an increased risk of type 1 diabetes autoimmunity compared with gluten introduction at >3 months of age. However, after 3 months of age, the age of gluten introduction had no effect on the risk of type 1 diabetes [14].

Studies that have evaluated the consumption of peanuts during lactation have shown a wide range of results from reduced to increased sensitization [26,47,48,49,50]. One study that evaluated maternal consumption of peanuts and tree nuts in the pre-pregnancy period found that a higher level of consumption in mothers who were not allergic to peanuts was associated with protection against peanut and tree nut allergy in the infant [51]. A recent 2018 study investigated the relationship between maternal consumption of peanuts during breastfeeding, the timing of direct introduction of peanuts, and peanut sensitisation at the age of seven years. Study results showed that maternal peanut consumption during breastfeeding combined with direct peanut introduction in the first year of life was associated with a lower risk of peanut sensitisation than all other maternal-infant consumption combinations [50].

The efficacy Is defined by the patient’s tolerance to the increase in amount over time, so that the tolerated amount could later be consumed in the basic diet [2]. Of course, immunotherapy is mainly used to increase the safe range of food consumption, as in many cases, the food allergy, at least to egg and cow’s milk, resolves before preschool age [2]. Allergen avoidance remains the safest solution, as the main ethical issue is the potential for life-threatening anaphylactic episodes in 10–35% of children, possibly due to co-factors unknown at the time of testing [52].

While it has been shown that maternal dietary intake during pregnancy and breastfeeding can, to some extent, influence immune markers in the child that may alter the risk of allergy development, the multiple variables of food antigens differ from person to person, so we must be careful to interpret these complex relationships clearly. Hence, the EAACI guidelines [2] recommendation not to supplement with cow’s milk, in order to prevent food allergy in newborns who are breastfed during the first week of life.

The majority of trials did not find a reduction in the prevalence of food allergy when women avoided food allergens during pregnancy and postpartum. Food allergens are not isolated, so avoiding a food group in a population at risk outweighs the benefits, as reducing nutrient and fiber intake negatively affects the health of women and infants.

## 8. Concluding Remarks

Breastfeeding is the most important protective nutrient for infant development and contributes to the maturation of defense mechanisms in their adult future lives. The preventive role of breastfeeding has a positive effect on allergy prevention, which is countered by the early introduction of solid foods, but large studies are needed to consolidate the evidence. Breastfeeding is effective in providing partial protection to infants.

In conclusion, all mothers, regardless of allergy risk, should be encouraged to avoid certain foods that can cause allergies in their infants while breastfeeding. Proper and balanced nutrition prior and during lactation will help improve breastmilk quality and help in addressing allergies more effectively or even negate them.

## Figures and Tables

**Table 1:** Effect of breastfeeding on allergic conditions.

Study	Allergic Condition	Effect of Breastfeeding
Amazouz et al., 2021 [19]	Asthma	Mediterranean diet of mother controls asthma
Klop et al., 2017 [20]	Asthma	Protective action against asthma for the first 3 years
Ehlayel et al., 2008 [21]	Asthma	protective against respiratory diseases, unlike formula feeding
Chui et al. [22]	Eczema	reducing eczema up to the age of 2 years
Kull et al., 2005 [40]	Eczema and allergic rhinitis	Breastfeeding for more than 4 months had a lower risk of eczema and allergic rhinitis at 4 years of age
Codispoti et al., 2010 [41]	Allergic rhinitis	Prolonged breastfeeding in African American patients reduced the risk of allergic rhinitis at the age of 3 years
Morales et al., 2012 [42]	Wheezing, lower respiratory infections and eczema	Exclusive breastfeeding for 4–6 months reduced wheezing, lower respiratory infections and eczema at 7–14 months
Storrø edt al., 2010 [37]	Wheezing, asthma and eczema	Longer duration of breastfeeding for at least 6 months was effective in reducing the incidence of eczema, asthma and wheezing at the ages of 1, 2 and 6 years
Adeyeye et al., 2019 [38]	Wheezing	Protective against wheezing
Han et al., 2019 [39]	Allergic rhinitis	Long term breast feeding (≥12 months) was associated with a reduced risk of allergic rhinitis
Järvinen et al., 2019 [4]	Allergic diarrhoea	Breastfeeding by allergen-sensitised mothers may help prevent allergic diarrhoea

**Table 2:** Guidelines and recommendations on solid food introduction while breastfeeding.

Study	Solid Food	Guidelines/Recommendations for Introduction
European Society for Paediatric Gastroenterology, Hepatology and Nutrition (ESPGHAN) [14]	Solid foods in general	4–6 months concurrently with breastfeeding
European Society for Paediatric Gastroenterology, Hepatology and Nutrition (ESPGHAN) [14]	Cows’s milk	Strong recommendation to start cow’s milk after the first year
European Academy of Allergy and Clinical Immunology (EAACI) [2]	Eggs, cow’s milk and peanuts	Eggs not raw, small quantities (½ egg) 2 times a week from the 4th month Not to supplement with cow’s milk, in order to prevent food allergy in newborns who are breastfed during the first week of life
European Academy of Allergy and Clinical Immunology (EAACI) [2]	Peanuts	Peanuts, initially in a safe form with slow administration so as not to block the airways. In local cusines with traditional use of peanuts, introduction from 4 to 11 months while following local guidelines
Paediatric Allergy Association [23]	Peanuts	Infants who are at high risk of developing a peanut allergy should be exposed to peanuts at an early age, following an appropriate expert assessment

## Data Availability

Not applicable.
